# Stillbirths including intrapartum timing: EN-BIRTH multi-country validation study

**DOI:** 10.1186/s12884-020-03238-7

**Published:** 2021-03-26

**Authors:** Kimberly Peven, Louise T. Day, Harriet Ruysen, Tazeen Tahsina, Ashish KC, Josephine Shabani, Stefanie Kong, Shafiqul Ameen, Omkar Basnet, Rajib Haider, Qazi Sadeq-ur Rahman, Hannah Blencowe, Joy E. Lawn, EN-BIRTH Study Group, Qazi Sadeq-ur Rahman, Ahmed Ehsanur Rahman, Tazeen Tahsina, Sojib Bin Zaman, Shafiqul Ameen, Tanvir Hossain, Abu Bakkar Siddique, Aniqa Tasnim Hossain, Tapas Mazumder, Jasmin Khan, Taqbir Us Samad Talha, Rajib Haider, Hafizur Md. Rahman, Anisuddin Ahmed, Shams Arifeen, Omkar Basnet, Avinash K. Sunny, Nishant Thakur, Regina Gurung, Anjani Kumar Jha, Bijay Jha, Ram Chandra Bastola, Rajendra Paudel, Asmita Paudel, Ashish KC, Nahya Salim, Donat Shamba, Josephine Shabani, Kizito Shirima, Meena Narcis Tarimo, Godfrey Mbaruku, Honorati Masanja, Louise T. Day, Harriet Ruysen, Kimberly Peven, Vladimir S. Gordeev, Georgia R. Gore-Langton, Dorothy Boggs, Stefanie Kong, Sarah Moxon, Hannah Blencowe, Angela Baschieri, Simon Cousens, Joy E. Lawn

**Affiliations:** 1 Maternal, Adolescent, Reproductive & Child Health (MARCH) Centre, London School of Hygiene & Tropical Medicine, Keppel Street, London, WC1E 7HT UK; 2 Florence Nightingale Faculty of Nursing, Midwifery & Palliative Care, King’s College London, London, UK; 3 Maternal and Child Health Division, International Centre for Diarrhoeal Disease Research, Bangladesh (iccdr,b), Dhaka, Bangladesh; 4 Department of Women’s and Children’s Health, Uppsala University, Uppsala, Sweden; 5 Department of Health Systems, Impact Evaluation and Policy, Ifakara Health Institute, Dar es Salaam, Tanzania; 6 Research Division, Golden Community, Lalitpur, Nepal

**Keywords:** Stillbirth, Birth, Neonatal, Maternal, Validity, Survey, Hospital records, Health management information systems

## Abstract

**Background:**

An estimated >2 million babies stillborn around the world each year lack visibility. Low- and middle-income countries carry 84% of the burden yet have the least data. Most births are now in facilities, hence routine register-recording presents an opportunity to improve counting of stillbirths, but research is limited, particularly regarding accuracy. This paper evaluates register-recorded measurement of hospital stillbirths, classification accuracy, and barriers and enablers to routine recording.

**Methods:**

The EN-BIRTH mixed-methods, observational study took place in five hospitals in Bangladesh, Nepal and Tanzania (2017–2018). Clinical observers collected time-stamped data on perinatal care and birth outcomes as gold standard. To assess accuracy of routine register-recorded stillbirth rates, we compared birth outcomes recorded in labour ward registers to observation data. We calculated absolute rate differences and individual-level validation metrics (sensitivity, specificity, percent agreement). We assessed misclassification of stillbirths with neonatal deaths. To examine stillbirth appearance (fresh/macerated) as a proxy for timing of death, we compared appearance to observed timing of intrauterine death based on heart rate at admission.

**Results:**

23,072 births were observed including 550 stillbirths. Register-recorded completeness of birth outcomes was > 90%. The observed study stillbirth rate ranged from 3.8 (95%CI = 2.0,7.0) to 50.3 (95%CI = 43.6,58.0)/1000 total births and was under-estimated in routine registers by 1.1 to 7.3 /1000 total births (register: observed ratio 0.9–0.7). Specificity of register-recorded birth outcomes was > 99% and sensitivity varied between hospitals, ranging from 77.7–86.1%. Percent agreement between observer-assessed birth outcome and register-recorded birth outcome was very high across all hospitals and all modes of birth (> 98%). Fresh or macerated stillbirth appearance was a poor proxy for timing of stillbirth. While there were similar numbers of stillbirths misclassified as neonatal deaths (17/430) and neonatal deaths misclassified as stillbirths (21/36), neonatal deaths were proportionately more likely to be misclassified as stillbirths (58.3% vs 4.0%). Enablers to more accurate register-recording of birth outcome included supervision and data use.

**Conclusions:**

Our results show these routine registers accurately recorded stillbirths. Fresh/macerated appearance was a poor proxy for intrapartum stillbirths, hence more focus on measuring fetal heart rate is crucial to classification and importantly reduction in these preventable deaths.

## Key findings

**Table:** 

**What is known and what is new about this study?**
• An estimated >2 million babies are stillborn each year by WHO’s international comparison definition of > 28 weeks’ gestation. 70% of births are in countries still reliant on population-based survey data to monitor health outcomes. Improving data in routine systems is vital to decreasing preventable deaths. • EN-BIRTH study used clinical observer-assessed data as the gold standard, and was the largest multi-country, multi-site study (*n* = 23,072 births, 550 stillbirths) to assess accuracy of register-recorded stillbirth rates in low- and middle-income countries (LMICs). The large sample size allowed examination of stillbirth timing and if measurement is affected by mode of birth. The qualitative component sought to explore the specific barriers and enablers to routine register recording of birth outcomes.
**Registers - what did we find and what does it mean?**
• Data completeness for birth outcomes in labour ward registers was high in all five hospitals, over 90%. • These routine registers under-estimated the observer-assessed stillbirth rate by 1.1 to 7.4 per 1000 total births. Recorded birth outcomes had high percent agreement (> 98%) and specificity (> 99%) with variable sensitivity (77.7–86.1%). • Hospitals with identical register design differed in completeness and accuracy. Qualitative findings suggest supervision, perceived usefulness of data and data culture contribute to improved quality of register data.
**Classification of stillbirths - what did we find?**
• We found proportionately more neonatal deaths on labour ward were misclassified as stillbirths than stillbirths misclassified as neonatal deaths. However, the absolute numbers of misclassified birth outcomes were similar in each direction (21 of 36 labour ward neonatal deaths misclassified as stillbirths and 17 of 430 stillbirths misclassified as neonatal deaths). • All registers used stillbirth appearance (fresh/macerated) to categorise stillbirth timing, however, this was not a good proxy since nearly one-third (31.1%) of observed intrapartum stillbirths were recorded as macerated. Most women (96.5%) had a fetal heart rate recorded on admission to the labour and delivery ward, which could be used to help identify ante/intrapartum stillbirths. • 91% of stillbirths met weight and/or gestational age cut-offs (ICD-10), but 9% were < 1000 g or < 28 weeks’ gestation. Gestational age was recorded for over 95% of stillbirths, however, whilst most stillbirths were weighed in three hospitals, < 27% were weighed in the two hospitals in Bangladesh.
**What next and research gaps?**
• Routine facility registers now reach almost 80% of the world’s births, we found most (70–90%) observed facility-stillbirths were accurately captured, however they are under-used for national and global accountability. • Reducing stillbirth/neonatal death misclassification requires devices and systems to easily measure and record heart rate as well as training in timely newborn care, recognising signs of life, and resuscitation to save more lives. • “Fresh/macerated” is widely used and recorded in registers to classify intrapartum/antepartum stillbirth, but this is inaccurate. An intentional focus on measuring and recording fetal heart rate on admission is crucial for every woman and her baby. • Register design, staff training, supervision and data culture could improve accuracy but more research is required on these, and also on flow in Health Management Information Systems (HMIS). • Linkages with civil and vital registration systems (birth/death certificates) and with Maternal and Perinatal Death Surveillance and Response (MPDSR) also hold potential but require implementation research. • Use of data and more innovation to address high intrapartum stillbirth rates is crucial. • Bereavement support is understudied in LMICs, but important to care for affected families, communities, and caregivers.

## Background

An estimated >2 million babies are stillborn each year, of which 84% are in low- and middle-income countries (LMICs), notably in south Asia and sub-Saharan Africa [1, 2]. Although the magnitude of global stillbirths is similar to neonatal deaths [3], stillbirths are not included in the sustainable development goals [4] and are absent from many health metrics including quality-adjusted life-years (QALYs), and disability-adjusted life-years (DALYs) [5, 6]. Yet stillbirths are associated with detrimental psychological effects for women, families and health workers as well as substantial direct and indirect economic costs [5]. Stillbirths continue to be omitted in political commitments—of 90 countries reporting on the *Every Newborn* Action Plan (ENAP), more than 80% have a target for neonatal mortality rate reduction, while 32% have for stillbirths [7]. Although evidence suggests that high coverage of 10 currently available interventions could prevent almost half of stillbirths [8], some health workers and many politicians do not perceive stillbirths as being preventable [9].

Definitions of stillbirth are often poorly understood or applied. International Classification of Diseases (ICD) recommends collecting data on all babies showing no signs of life with a birthweight of 500 g or more [10]. For international statistical comparison, stillbirths are defined as death of a fetus before birth weighing 1000 g or more and are reported as a rate per 1000 total births (live births plus stillbirths). Where birthweight is not known, ICD recommends using a gestational age threshold of 22 or more weeks for recording and 28 or more weeks for international comparisons. However, a review of these definitions is currently underway and, in line with recent global stillbirth estimates, the use of gestational age at birth in preference to birthweight criteria is likely to be recommended [2]. Whilst systematic global reviews found stillbirth rate data for over 147 countries, often these data are not used in national or global policy and planning [11].

Population-based surveys remain the major source of information on stillbirths from LMICs. These can provide population-level information especially where routine health system data are weak and also capture births outside health facilities. The Demographic and Health Survey Program (DHS) is the largest system of household surveys, covering over 90 countries. DHS changed in 2020 from using a birth history only on live births, to a full pregnancy history in order to improve the capture of stillbirths, based on a randomised comparison of these two approaches [12, 13]. However, even with the pregnancy history approach, there are challenges that frequently lead to under-capture of stillbirths. Additionally, nationally representative surveys are only conducted approximately every 2-5 years and are costly.

Now, with nearly 80% of births worldwide in facilities [14], facility data through health management information systems (HMIS) have the potential to improve the monitoring of stillbirth outcomes. However, in many countries, routine facility registers, which are the primary data source for HMIS, are not trusted or used for data collection on birth complications and stillbirths [15].

In addition to tracking stillbirth rates, being able to identify the intrapartum stillbirth rate is imperative to help address the large number of preventable deaths that occur during labour and birth [11, 16, 17]. Yet even when stillbirth data are reported through the system up to the national level, usually only overall stillbirth rates are included [11]. In LMICs, the most common approach used in facility registers is the appearance of the stillborn baby, taking fresh stillbirth as a surrogate of intrapartum stillbirth, and macerated as a surrogate of antepartum stillbirth. The assumption is that a fresh stillbirth died within 12 h or less of birth, most likely during labour [11].

Routine facility-based recording and timely reporting of stillbirths have the potential to increase visibility and drive change [18, 19], yet previous validation research has mainly focussed on verbal autopsy. *Every Newborn*, agreed by all United Nations member states and > 80 development partners, includes an ambitious measurement improvement roadmap [20, 21] with an urgent focus on validating indicators for care and outcomes around the time of birth.

As part of this roadmap, the *Every Newborn*– Birth Indicators Research Tracking in Hospitals (EN-BIRTH) study aimed to validate selected newborn and maternal indicators for routine facility-based tracking of coverage, quality of care, and outcomes [22, 23].

## Objectives

This paper is part of a supplement based on the EN-BIRTH multi-country validation study, “*Informing measurement of coverage and quality of maternal and newborn care*” and focuses on stillbirth with five objectives:

1. **Determine NUMERATOR accuracy/validity** for register-recorded hospital stillbirth rate compared to observation data.
2. **Assess MISCLASSIFICATION of stillbirth** both as neonatal deaths and those who did not meet gestational age/birthweight cut-offs (early fetal loss).
3. **Compare classification of stillbirth TIMING** for confirmed intrapartum stillbirth and register-recorded stillbirth appearance (fresh/macerated).
4. **Analyse GAPS** in measuring coverage and quality of care for stillbirths.
5. **Evaluate BARRIERS AND ENABLERS** to routine labour ward register-recording of stillbirths.

## Methods

EN-BIRTH was a mixed-methods observational study comparing data from clinical observers (considered gold standard) to survey-reported and register-recorded coverage of perinatal care and perinatal outcomes. Detailed information regarding the research protocol and methods has been published separately [22, 23]. In summary, data were collected from June 2017 to July 2018 in five public secondary/tertiary hospitals in three high burden countries: Maternal and Child Health Training Institute, Azimpur and Kushtia General Hospital in Bangladesh (BD); Pokhara Academy of Health Sciences in Nepal (NP); Temeke Regional Hospital and Muhimbili National Referral Hospital in Tanzania (TZ) (Additional file 1). Participants were consenting women admitted to labour and delivery wards in the five study hospitals. To avoid maternal distress, data collectors were trained to consent women admitted in labour with a live fetus, and women with a prior diagnosis of intrauterine death were excluded. In some cases where a fetal heart rate was not obtained on admission, women were still included in the study. Trained clinical researchers observed participants 24 h per day and recorded data on care and outcomes, including stillbirth and neonatal death, as the external gold standard. Observers recorded birth outcomes (live/stillbirth) as well as stillbirth appearance (fresh/macerated). Trained data extractors captured information from existing hospital registers. Interviewers did not ask women with stillbirths or neonatal deaths about birth outcomes to minimise risk of emotional trauma, so this paper focuses on validation of register-recorded outcomes compared to observer-assessed outcomes. All data were collected with a custom-built android tablet-based software application, including timestamps for observation data [22]. Health workers and data collectors were interviewed about barriers and enablers to use of routine registers in recording perinatal care and outcomes. Results are reported in accordance with the STROBE Statement checklist for cross-sectional studies (Additional file 2).

Quantitative analysis was undertaken using R version 3.6.1 [24].

### Objective 1: Numerator validation

**Fig. 1:**
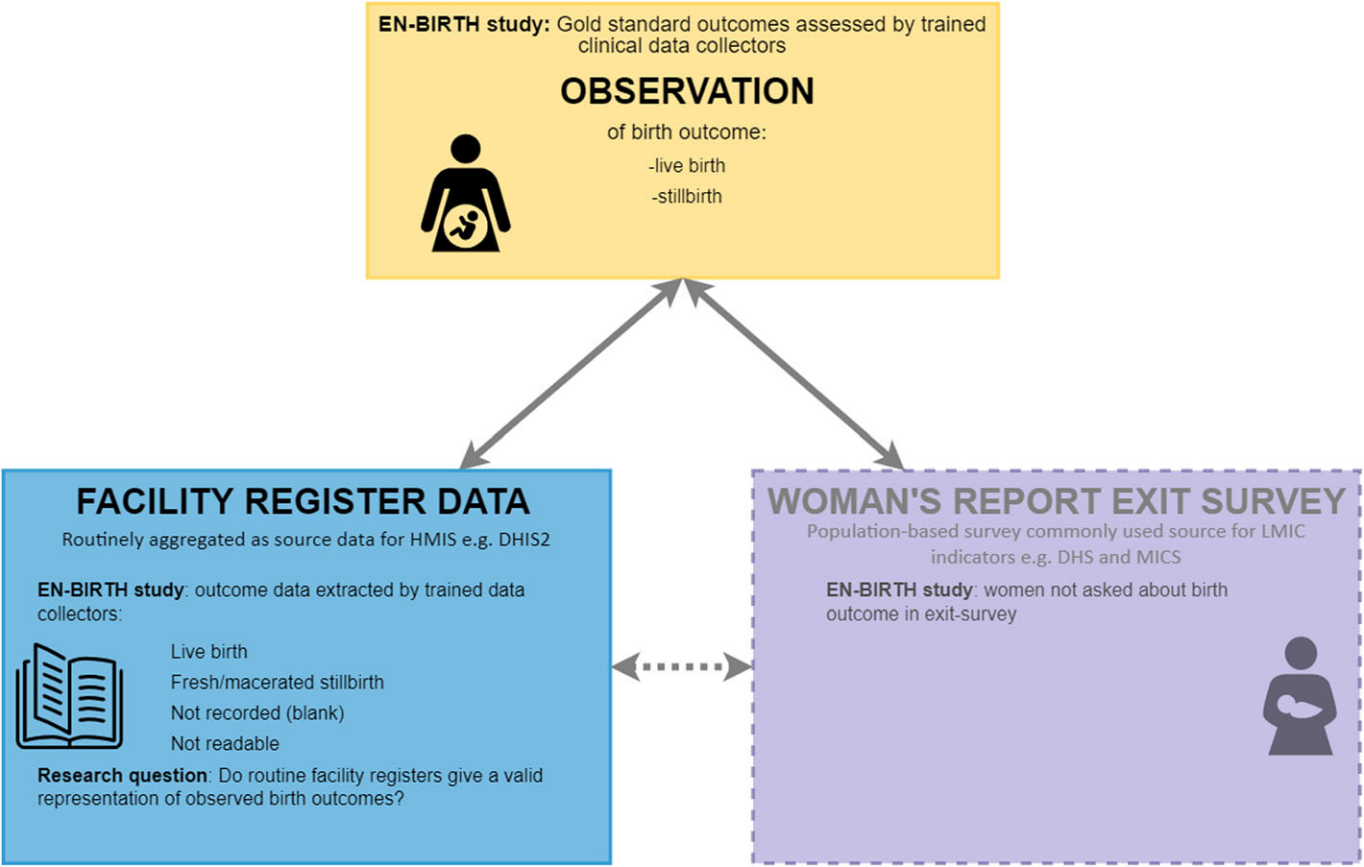
Stillbirth: validation design, EN-BIRTH study. EN-BIRTH validation design comparing observation gold standard with register-recorded and women’s report on exit survey; EN-BIRTH data collection tools (observation checklist, register data extraction tool and exit survey tool) are published separately [22]

We compared routine register-recorded birth outcomes to gold standard observer-assessed birth outcomes (live birth or stillbirth), stratifying by hospital and mode of birth (vaginal births and caesarean births) (Fig. 1). We calculated absolute differences in study stillbirth rates to determine under- or over-estimate of register records compared to observer-assessed outcomes. Similar to verification ratios in data quality review (DQR) methods [25], we calculated validity ratios (register rate: observed rate), heat-mapping results using standard DQR cut-offs (over/underestimate by 0–5%, 5–10%, 10–15%, 15–20% and > 20%).

**Fig. 2:**
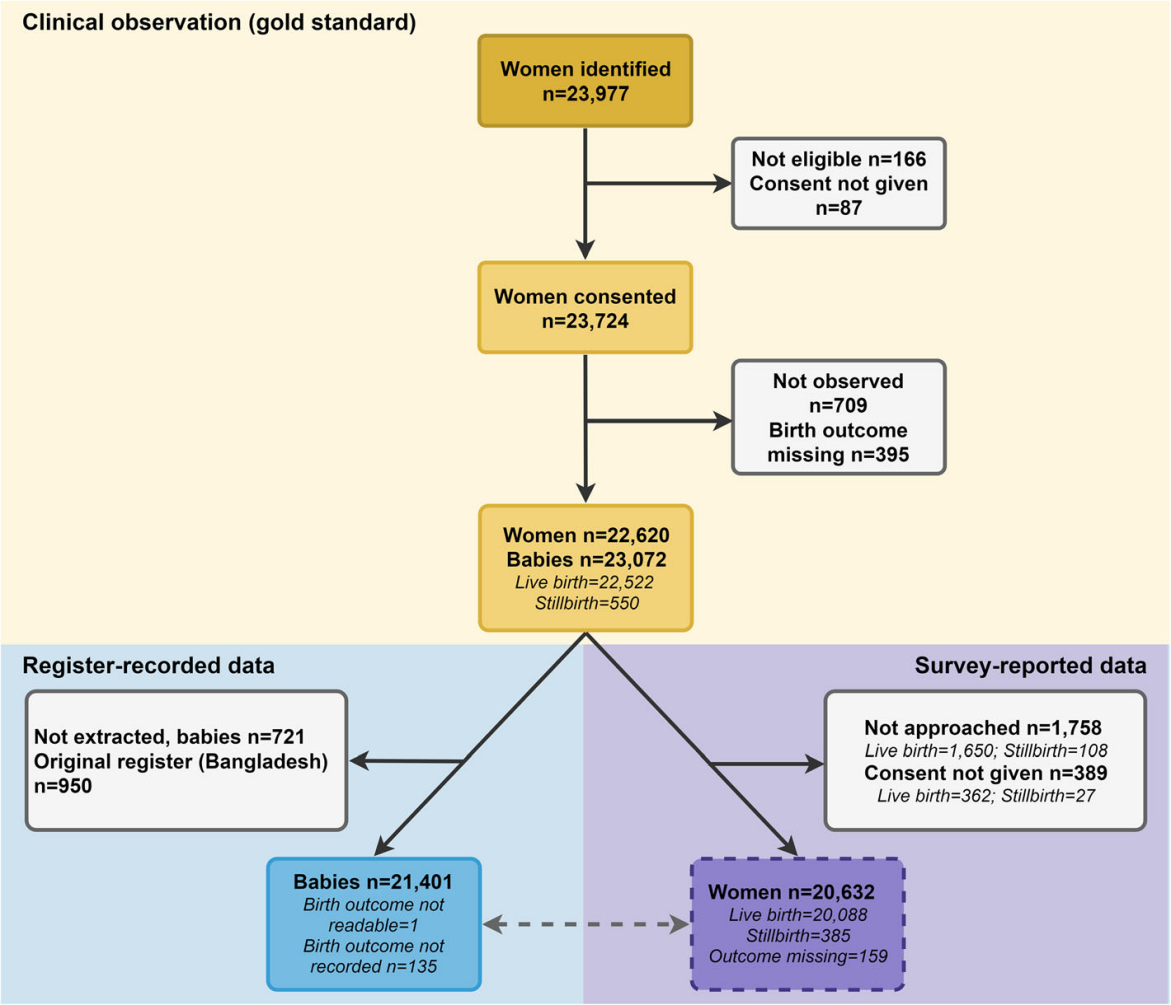
Flow diagram for stillbirths, EN-BIRTH study (*n* = 23,072)

As low prevalence of stillbirth affected individual-level validity “diagnostic test” methods (low cell counts in two-by-two tables), we report percent agreement for all sites and modes of birth. Where two-by-two tables contained cell counts of 10 or more in each column, we calculated sensitivity and specificity of register-recorded stillbirth to measure observed stillbirth. Area under the curve (AUC), inflation factor (IF), positive predictive value (PV), and negative predictive value (NPV) were also calculated.

The data quality dimension of completeness was calculated. Typically register data are aggregated from documented events e.g. stillbirth outcome [26], so where register-recorded birth outcome was incomplete (blank), we assumed these babies would have been counted as live-born. We also calculated validity statistics excluding these cases.

We combined hospital-specific validity results using a random effects meta-analysis approach and used I^2^ and τ^2^ to assess heterogeneity between hospitals. To determine the reliability of the observational data (gold standard), supervisors duplicated observation (and register data extraction) for a subset of 5% for which we calculated Cohen’s Kappa coefficients and percent agreement.

### Objective 2: Stillbirth misclassification

For babies with both observer-assessed birth outcomes and register-recorded data, we compared register-recorded birth outcome for all observed stillbirths and neonatal deaths occurring on the labour ward and calculated the proportion of stillbirths misrecorded as neonatal deaths and neonatal deaths misrecorded as stillbirths.

We evaluated the gestational age extracted from medical records or women’s report and observed birthweight for stillbirths in relation to ICD-11 definitions for international comparison [10]. We examined the percent distribution of stillbirths by birthweight and by gestational age.

### Objective 3: Stillbirth classification

We considered a stillborn baby as a confirmed intrapartum stillbirth if fetal heart sounds were checked and present on admission to labour and delivery ward. Where the fetal heart rate was not checked on admission or the admission heart rate was not recorded or marked as absent the baby was not included in intrapartum stillbirth analysis. We examined the proportion of these intrapartum stillbirths recorded in the register as fresh or macerated stillbirths, to understand the accuracy of register-recorded stillbirth appearance (fresh/macerated) as a proxy for timing of intrauterine death.

### Objective 4: Gap analysis

We analysed gaps in observed coverage of three immediate routine practices for newborn babies (drying, wrapping, and weighing) for both live births and stillbirths (fresh and macerated) and compared these to exit survey report examining gaps and levels of “don’t know” responses.

### Objective 5: Barriers and enablers to routine recording

As part of the wider EN-BIRTH study, focus group discussions and in-depth qualitative interviews were conducted to understand the barriers and enablers to the use of routine registers in recording various aspects of perinatal care and outcomes [27]. Detailed qualitative methods and overall results are available in an associated paper [27]. In summary, we purposively sampled two groups of respondents: hospital health workers providing perinatal care in EN-BIRTH sites (nurses/midwives/doctors) and data collectors involved in the EN-BIRTH study (clinical observers/data extractors/supervisors). At least two in-depth interviews were conducted for each group in each hospital (Additional file 3). Semi-structured in-depth interview guides and semi-structured focus group guides were developed based on the Performance of Routine Information System Management (PRISM) conceptual framework [28]. We conducted a secondary analysis of the EN-BIRTH qualitative data to identify themes related to recording of birth outcomes in routine hospital registers. This paper specifically presents themes relating to recording of birth outcomes.

## Results

Across the five participating hospitals, 23,977 women were identified on admission to the labour and delivery ward. Of those, 23,811 met eligibility criteria, 23,724 consented to participate. Seven hundred nine were not observed and 395 birth outcomes were missing. Included in this analysis were 22,620 women and 23,072 births (849 twins, 42 triplets). Data extraction from the registers was completed for 21,401 (92.8%) births (Fig. 2).

Table 1 shows characteristics of the EN-BIRTH study sample by site and birth outcome. Among live births, 84.6% weighed over 2500 g and just 1.2% were under 1500 g, among stillbirths 16.8% were under 1500 g. While birthweight was missing for just 2.0% of live births, birthweight was missing for nearly one-third (31.1%) of stillbirths, highest in Bangladesh (Azimpur 72.7, Kushtia 90.5%). Similarly, while the sex of the baby was missing for just 0.3% of live births, this was missing for 6.4% of stillbirths. Over three-quarters (76.8%) of live births were 37 or more weeks gestation on admission, 38.0% of stillbirths were term or post term on admission.

**Table 1:** Characteristics of live births and stillbirths in labour and delivery wards, EN-BIRTH Study (*n* = 23,072 births)

	Bangladesh		Bangladesh		Nepal		Tanzania		Tanzania		All sites	
	Azimpur Tertiary		Kushtia District		Pokhara Regional		Temeke Regional		Muhimbili National			
	Live births	Stillbirths	Live births	Stillbirths	Live births	Stillbirths	Live births	Stillbirths	Live births	Stillbirths	Live births	Stillbirths
	n (%)	n (%)	n (%)	n (%)	n (%)	n (%)	n (%)	n (%)	n (%)	n (%)	n (%)	n (%)
**a) Total babies observed**^**a**^	2896	11	2308	74	7175	126	6634	153	3509	186	22,522	550
**Mode of birth**												
Normal vaginal birth	760 (26.2)	10 (90.9)	1337 (57.9)	51 (68.9)	5789 (80.7)	98 (77.8)	6157 (92.8)	133 (86.9)	1468 (41.8)	118 (63.1)	15,511 (68.9)	410 (74.4)
Vaginal breech/ Vacuum/ Forceps	1 (0)	0 (0)	0 (0)	0 (0)	337 (4.7)	13 (10.3)	9 (0.1)	1 (0.7)	8 (0.2)	2 (1.1)	355 (1.6)	16 (2.9)
Caesarean Section	2135 (73.7)	1 (9.1)	971 (42.1)	23 (31.1)	1049 (14.6)	14 (11.1)	467 (7)	17 (11.1)	2033 (57.9)	61 (32.6)	6655 (29.6)	116 (21.1)
Missing	0 (0)	0 (0)	0 (0)	0 (0)	0 (0)	1 (0.8)	1 (0)	2 (1.3)	0 (0)	6 (3.2)	1 (0)	9 (1.6)
**Birthweight**												
< 1000	1 (0)	0 (0)	6 (0.3)	1 (1.4)	7 (0.1)	20 (15.9)	13 (0.2)	0 (0)	23 (0.7)	21 (11.5)	50 (0.2)	42 (7.7)
1000–1499	1 (0)	0 (0)	27 (1.2)	0 (0)	23 (0.3)	15 (11.9)	14 (0.2)	8 (5.2)	132 (3.8)	27 (14.8)	197 (0.9)	50 (9.1)
1500–2499	351 (12.1)	0 (0)	434 (18.8)	3 (4.1)	795 (11.1)	35 (27.8)	440 (6.6)	26 (17)	737 (21)	57 (31.1)	2757 (12.3)	121 (22.1)
2500+	2525 (87.2)	3 (27.3)	1801 (78.2)	3 (4.1)	6233 (86.9)	41 (32.5)	5968 (90.1)	83 (54.2)	2515 (71.7)	34 (18.6)	19,042 (84.6)	164 (30)
Birthweight missing	18 (0.6)	8 (72.7)	36 (1.6)	67 (90.5)	115 (1.6)	15 (11.9)	188 (2.8)	36 (23.5)	99 (2.9)	44 (24.1)	456 (2.0)	170 (31.1)
**Gestational age** (recorded at admission)												
< 28 weeks	1 (0)	0 (0)	6 (0.3)	1 (1.4)	6 (0.1)	22 (17.5)	1 (0)	2 (1.3)	12 (0.3)	7 (3.8)	26 (0.1)	32 (5.8)
28–32 weeks	1 (0)	1 (9.1)	35 (1.5)	5 (6.8)	20 (0.3)	12 (9.5)	45 (0.7)	7 (4.6)	186 (5.3)	53 (28.6)	287 (1.3)	78 (14.2)
32–36 weeks	637 (22)	4 (36.4)	464 (20.1)	43 (58.1)	337 (4.7)	27 (21.4)	1479 (22.3)	51 (33.6)	971 (27.7)	79 (42.7)	3888 (17.3)	204 (37.2)
37+	2180 (75.3)	5 (45.5)	1789 (77.5)	25 (33.8)	6550 (91.4)	57 (45.2)	4503 (67.9)	78 (51.3)	2267 (64.7)	43 (23.2)	17,289 (76.8)	208 (38)
Missing	77 (2.7)	1 (9.1)	13 (0.6)	0 (0)	257 (3.6)	8 (6.3)	605 (9.1)	14 (9.2)	69 (2)	3 (1.6)	1021 (4.5)	26 (4.7)
**Sex**												
Female	1437 (49.6)	4 (36.4)	1118 (48.4)	36 (48.6)	3315 (46.2)	54 (42.9)	3201 (48.3)	64 (41.8)	1723 (49.1)	90 (48.4)	10,794 (47.9)	248 (45.1)
Male	1458 (50.3)	7 (63.6)	1189 (51.5)	31 (41.9)	3837 (53.5)	66 (52.4)	3397 (51.2)	84 (54.9)	1760 (50.2)	73 (39.2)	11,641 (51.7)	261 (47.5)
Ambiguous	1 (0)	0 (0)	1 (0)	3 (4.1)	13 (0.2)	0 (0)	7 (0.1)	0 (0)	3 (0.1)	3 (1.6)	25 (0.1)	6 (1.1)
Missing	0 (0)	0 (0)	0 (0)	4 (5.4)	10 (0.1)	6 (4.8)	29 (0.4)	5 (3.3)	23 (0.7)	20 (10.8)	62 (0.3)	35 (6.4)
**b) Total women observed**^**b**^	2872	11	2265	71	7111	119	6521	145	3331	174	22,100	520
**Women’s age**												
< 18 years	25 (0.9)	0 (0)	2 (0.1)	0 (0)	303 (4.3)	6 (5)	25 (0.4)	1 (0.7)	6 (0.2)	1 (0.6)	361 (1.6)	8 (1.5)
18–19 years	465 (16.2)	3 (27.3)	185 (8.2)	6 (8.5)	800 (11.3)	10 (8.4)	748 (11.5)	10 (6.9)	147 (4.4)	7 (4.0)	2345 (10.6)	36 (6.9)
20–24 years	1147 (39.9)	4 (36.4)	881 (38.9)	28 (39.4)	2978 (41.9)	37 (31.1)	2239 (34.3)	43 (29.7)	674 (20.2)	40 (23)	7919 (35.8)	152 (29.2)
25–29 years	854 (29.7)	3 (27.3)	700 (30.9)	26 (36.6)	2041 (28.7)	32 (26.9)	1636 (25.1)	41 (28.3)	1068 (32.1)	47 (27)	6299 (28.5)	149 (28.7)
30–34 years	294 (10.2)	1 (9.1)	353 (15.6)	8 (11.3)	785 (11)	20 (16.8)	1108 (17)	25 (17.2)	864 (25.9)	40 (23)	3404 (15.4)	94 (18.1)
35+ years	87 (3)	0 (0)	144 (6.4)	3 (4.2)	204 (2.9)	14 (11.8)	765 (11.7)	25 (17.2)	572 (17.2)	39 (22.4)	1772 (8.0)	81 (15.6)
**Women’s education**												
No education	37 (1.3)	0 (0)	70 (3.1)	5 (7)	256 (3.6)	7 (5.9)	196 (3)	3 (2.1)	62 (1.9)	3 (1.7)	621 (2.8)	18 (3.5)
Primary incomplete	110 (3.8)	0 (0)	117 (5.2)	7 (9.9)	240 (3.4)	9 (7.6)	76 (1.2)	1 (0.7)	40 (1.2)	5 (2.9)	583 (2.6)	22 (4.2)
Primary complete	333 (11.6)	2 (18.2)	317 (14)	18 (25.4)	287 (4)	8 (6.7)	28 (0.4)	1 (0.7)	4 (0.1)	1 (0.6)	969 (4.4)	30 (5.8)
Secondary incomplete	973 (33.9)	4 (36.4)	900 (39.7)	27 (38)	1582 (22.2)	25 (21)	3921 (60.1)	87 (60)	1189 (35.7)	77 (44.3)	8565 (38.8)	220 (42.3)
Secondary complete or higher	1259 (43.8)	5 (45.5)	828 (36.6)	13 (18.3)	4370 (61.5)	49 (41.2)	2268 (34.8)	52 (35.9)	2022 (60.7)	88 (50.6)	10,747 (48.6)	207 (39.8)
Missing	160 (5.6)	0 (0)	33 (1.5)	1 (1.4)	376 (5.3)	21 (17.6)	32 (0.5)	1 (0.7)	14 (0.4)	0 (0)	615 (2.8)	23 (4.4)
**Parity**												
Nullipara	1326 (46.2)	10 (90.9)	960 (42.4)	28 (39.4)	4255 (59.8)	58 (48.7)	2822 (43.3)	56 (38.6)	1270 (38.1)	62 (35.6)	10,633 (48.1)	214 (41.2)
Multipara	1493 (52)	1 (9.1)	1300 (57.4)	43 (60.6)	2849 (40.1)	61 (51.3)	3686 (56.5)	88 (60.7)	2057 (61.8)	112 (64.4)	11,385 (51.5)	305 (58.7)
Missing	53 (1.8)	0 (0)	5 (0.2)	0 (0)	7 (0.1)	0 (0)	13 (0.2)	1 (0.7)	4 (0.1)	0 (0)	82 (0.4)	1 (0.2)

^a^Data from observation

^b^Data collected from women’s registration and survey report

Less than one in ten women did not complete primary education (7.7% of stillbirths, 5.4% of live births). Two-thirds of births (68.9% of live births, 74.4% of stillbirths) were normal vaginal births. The stillbirth caesarean section rate was 21.1% (ranging from 9.1% in Azimpur, BD to 31.1% in Kushtia, BD and Muhimbili, TZ), two-thirds of the live birth caesarean section rate, 29.6% (ranging from 7.0% in Temeke, TZ to 73.7% in Azimpur, BD).

### Assessing biases in the data

Duplicate case observation inter-rater reliability showed high/substantial agreement for observed birth outcome (> 0.71). Register extraction percent agreement was high (>98%), however, kappa scores were lower, ranging from 0.69–1.00 (Additional file 4).

### Objective 1: Numerator validation

**Fig. 3:**
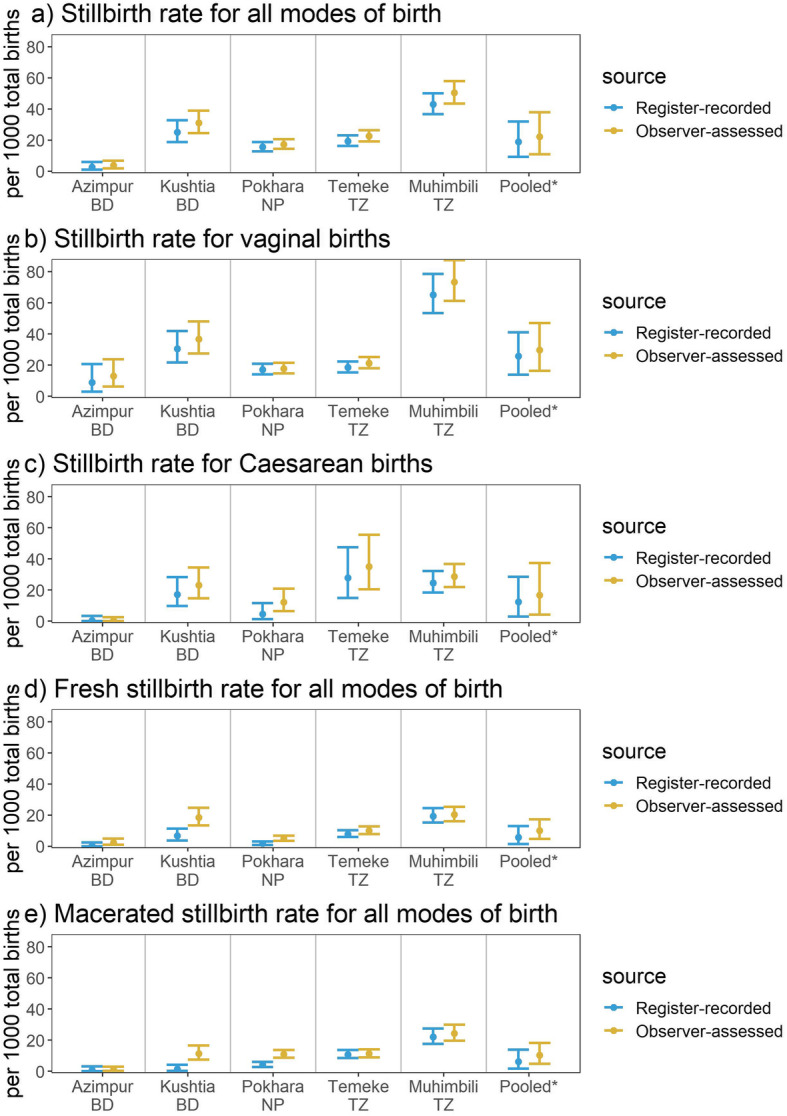
Stillbirth rates measured by observation and registers (95%CI), EN-BIRTH study (*n* = 23,072). *Random effects; Observed births *n* = 23,072; Register-recorded births *n* = 21,401; BD = Bangladesh, NP = Nepal, TZ = Tanzania

Across all sites, the observer-assessed study stillbirth rate was 22.3 (95%CI: 10.8,37.9) per 1000 total births. The register-recorded rate was slightly lower, 18.8 (95%CI: 9.2,31.8) per 1000 total births (Table 2 and Fig. 3). The observed study stillbirth rate ranged from 3.8 (95%CI: 2.0,7.0) per 1000 total births in Azimpur, BD to 50.3 (95%CI: 43.6,58.0) per 1000 total births in Muhimbili, TZ. Amongst vaginal births the study stillbirth rate ranged from 13.0 (95%CI: 6.6,24.6) in Azimpur to 73.5 (95%CI: 61.4,87.7) in Muhimbili, TZ and amongst caesarean births it ranged from 0.5 (95%CI: 0.0,3.0) in Azimpur to 35.1 (95%CI: 21.2,56.8) in Temeke, TZ. For both vaginal and caesarean births, the register-recorded stillbirth rate slightly under-estimated the observed rate.

**Table 2:** Individual-level validation of register measurement for stillbirth, EN-BIRTH study (*n* = 23,072)

	Bangladesh	Bangladesh	Nepal	Tanzania	Tanzania	All sites
	Azimpur Tertiary	Kushtia District	Pokhara Regional	Temeke Regional	Muhimbili National	Pooled (random effects)
**A) All modes of birth**						
Observed stillbirth rate (per 1000 total births, 95%CI)	3.8 (2.0,7.0)	31.1 (24.6,39.1)	17.3 (14.5,20.6)	22.5 (19.2,26.4)	50.3 (43.6,58)	22.3 (10.8,37.9)
Register recorded stillbirth rate (per 1000 total births, 95%CI)	2.7 (1.1,6.2)	25 (18.9,32.9)	15.6 (12.8,18.9)	19.2 (16.1,22.9)	43 (36.7,50.2)	18.8 (9.2,31.8)
No birth outcome recorded %	0.04 (0,0.29)	9.04 (7.86,10.38)	1.11 (0.88,1.39)	0.26 (0.16,0.42)	1.56 (1.19,2.03)	1.49 (0.22,3.86)
Register birth outcome not readable %	0.04 (0,0.29)	0 (0,0.23)	0.01 (0,0.09)	0.02 (0,0.10)	0.05 (0.01,0.22)	0.03 (0.01,0.06)
Sensitivity % (95% CI)	*	83.3 (71.5,91.7)	86.1 (78.4,91.8)	77.6 (69.9,84)	77.7 (70.8,83.5)	80.3 (76.5,83.8)
Specificity % (95% CI)	*	99.9 (99.6100)	99.9 (99.8,99.9)	99.8 (99.7,99.9)	99.5 (99.2,99.7)	99.8 (99.7,99.9)
Percent agreement	99.9	99.4	99.7	99.3	98.4	99.4 (98.9,99.8)
**B) Vaginal births**						
Observed stillbirth rate (per 1000 total births, 95%CI)	13 (6.6,24.6)	36.7 (27.7,48.4)	17.8 (14.7,21.5)	21.3 (17.9,25.2)	73.5 (61.4,87.7)	29.7 (16.3,47.0)
Register recorded stillbirth rate (per 1000 total births, 95%CI)	8.9 (3.3,21.8)	30.6 (21.9,42.3)	17.1 (14.1,20.8)	18.5 (15.4,22.3)	65.1 (53.7,78.7)	25.7 (13.9,41.0)
No birth outcome recorded %	0 (0,0.84)	6.19 (4.93,7.74)	0.28 (0.17,0.46)	0.23 (0.13,0.4)	0.76 (0.41,1.36)	0.86 (0.11,2.29)
Register birth outcome not readable %	0 (0,0.84)	0 (0,0.39)	0 (0,0.08)	0.02 (0,0.11)	0 (0,0.3)	0.01 (0.00,0.04)
Sensitivity % (95% CI)	*	89.7 (75.8,97.1)	88.8 (81.2,94.1)	79.2 (71.2,85.8)	83.2 (75,89.6)	84.6 (79.8,88.9)
Specificity % (95% CI)	*	99.8 (99.4100)	99.9 (99.7,99.9)	99.8 (99.7,99.9)	99.4 (98.8,99.7)	99.8 (99.6,99.9)
Percent agreement	100	99.5	99.7	99.4	98.2	99.4 (99.0,99.8)
**C) Caesarean births**						
Observed stillbirth rate (per 1000 total births, 95%CI)	0.5 (0,3)	23.1 (15.1,35.1)	12.2 (6.8,21.4)	35.1 (21.2,56.8)	28.7 (22.1,37)	16.8 (4.2,37.5)
Register recorded stillbirth rate (per 1000 total births, 95%CI)	0.6 (0,3.9)	17.3 (10.1,29)	4.6 (1.5,12.7)	28 (15.6,48.7)	24.7 (18.6,32.6)	12.5 (2.9,28.5)
No birth outcome recorded %	0.06 (0,0.39)	13.02 (10.89,15.48)	6.83 (5.28,8.77)	0.65 (0.17,2.04)	2.18 (1.61,2.93)	3.20 (0.39,8.55)
Register birth outcome not readable %	0.06 (0,0.39)	0 (0,0.55)	0.12 (0.01,0.75)	0 (0,1.02)	0.1 (0.02,0.39)	0.09 (0.02,0.18)
Sensitivity % (95% CI)	*	71.4 (47.8,88.7)	*	73.3 (44.9,92.2)	73.7 (60.3,84.5)	71.3 (62.1,79.7)
Specificity % (95% CI)	*	100 (99.6,100)	*	99.6 (98.4,99.9)	99.6 (99.2,99.8)	99.8 (99.6,100.0)
Percent agreement	99.9	99.3	99.8	98.7	98.8	99.4 (98.8,99.8)

*Validity statistics not shown where < 10 count in either column of two-by-two table

Observed: all modes of birth *n* = 23,072, vaginal births *n* = 16,284, caesarean birth *n* = 6769; Register: all modes of birth *n* = 21,401, vaginal births *n* = 15,458, and caesarean births *n* = 5926

Completeness for birth outcome in routine labour ward registers was high across all hospitals, ranging from 90.96% in Kushtia, BD to 99.96% in Azimpur, BD. Over 99% of all birth outcomes in registers were readable. Percent agreement between observer-assessed birth outcome and register-recorded birth outcome (Table 2) was very high across all hospitals and all modes of birth (> 98%). When other validity metrics could be calculated, specificity was very high, > 99%. Sensitivity was higher for vaginal births than caesarean births, ranging from 71.4% (95%CI: 47.8,88.7) for caesarean births in Kushtia, BD to 89.7% (95%CI:75.8,97.1) for vaginal births in Kushtia, BD. Additional validity measurements (AUC, IF, PPV, NPV) and two-way tables can be found in Additional files 5 and 6.

**Fig. 4:**
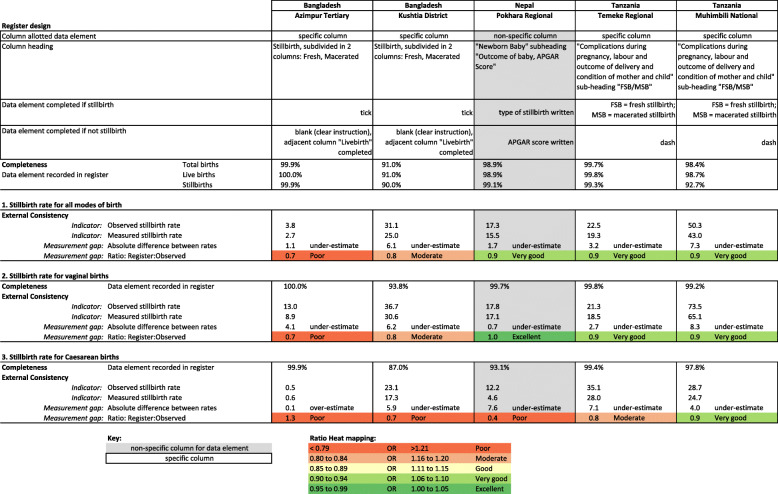
Routine register design and data quality dimensions for stillbirths by site, EN-BIRTH study. Observed births *n* = 23,072; Register-recorded births *n* = 21,401

Labour ward register design for each hospital is shown in Fig. 4 with absolute difference between observed and register-recorded study stillbirth rate, and validity ratios (register recorded rate: observed rate), heat mapped using Data Quality Review (DQR) 5, 10 and 20% cut-offs. Registers under-estimated the stillbirth rate by 1.1 (Azimpur, BD) to 7.3 (Muhimbili, TZ) per 1000 total births, with larger absolute differences occurring in hospitals with larger numbers of stillbirths. Validity ratios ranged from poor (0.7) and moderate (0.8) in Bangladesh to very good (0.9) in Nepal and Tanzania. Registers performed worse for caesarean sections for all hospitals except Muhimbili, TZ where the validity ratio was the same between vaginal and caesarean births.

### Objective 2: Stillbirth misclassification

**Fig. 5:**
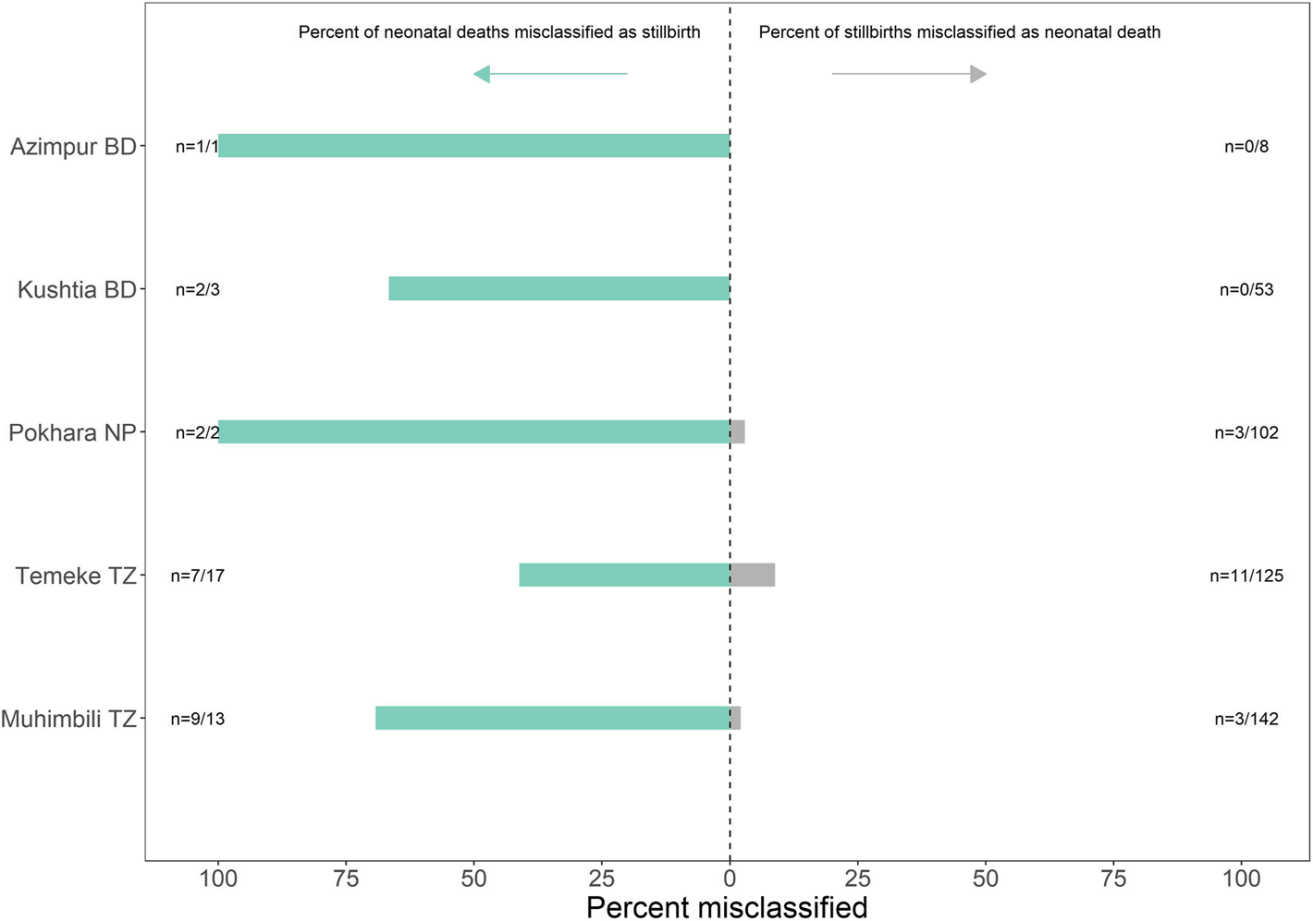
Proportion of stillbirths misclassified as deaths, proportion of labour ward neonatal deaths misclassified as stillbirths. BD = Bangladesh, NP = Nepal, TZ = Tanzania

Figure 5 shows the proportion of neonatal deaths (on labour ward) who were misclassified and recorded as stillbirths, as well as the proportion of stillbirths misclassified and recorded as neonatal deaths (for births with both observed and register-recorded birth outcomes). Of 36 neonatal deaths on the labour ward, over half (*n* = 21) were register-recorded as stillbirths. Of 430 stillbirths, 17 were recorded as neonatal deaths (4.0%).

**Fig. 6:**
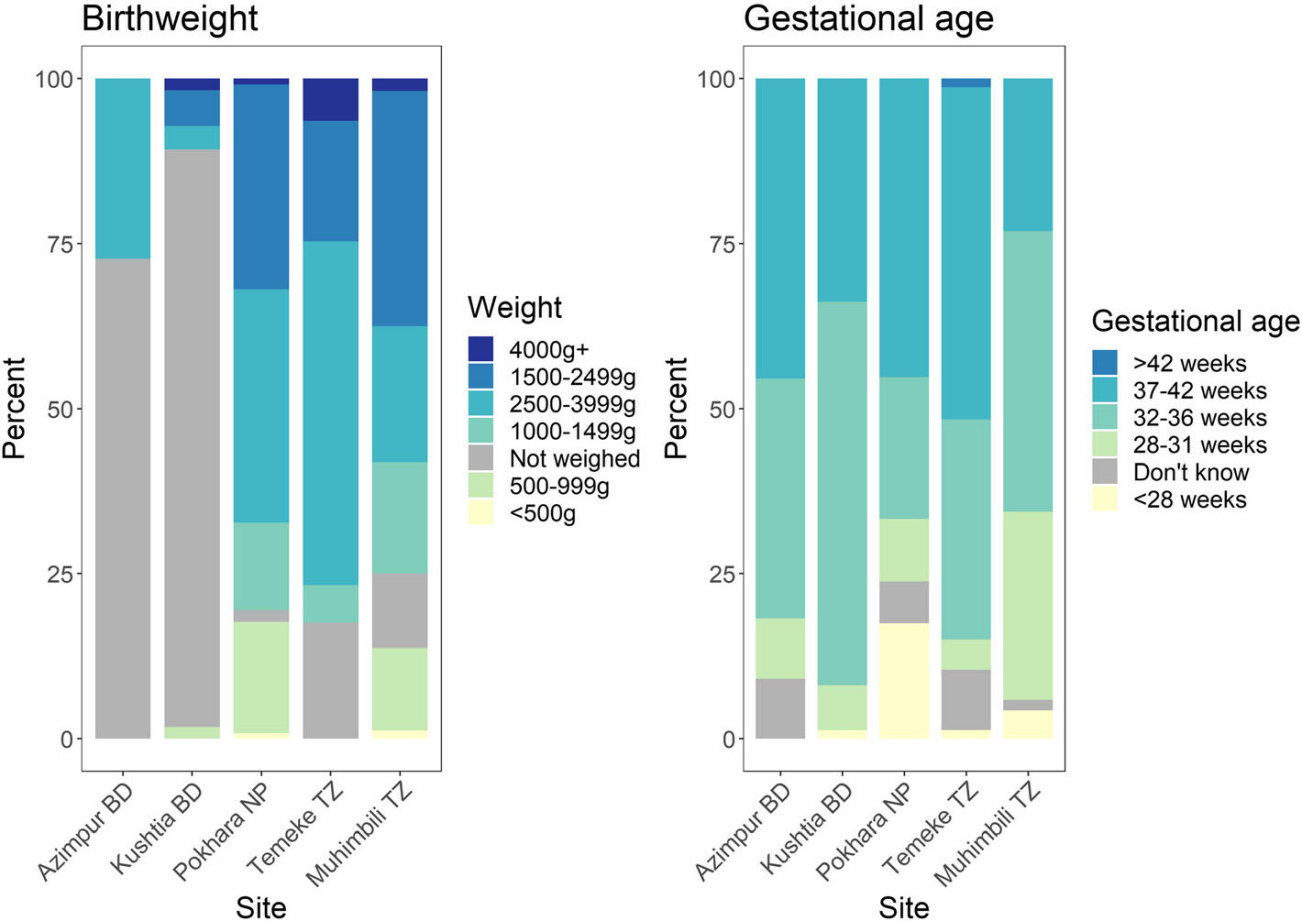
Percent distribution of stillbirths by birthweight (*n* = 482) and gestational age at admission (*n* = 550), EN-BIRTH study. From observation data; BD = Bangladesh, NP = Nepal, TZ = Tanzania

Most observer-assessed stillbirths were weighed, apart from the Bangladesh sites. Most stillbirths who were weighed, were over 1000 g. Less than 9% (*n* = 43) of observer-assessed stillbirths weighed under 1000 g (Fig. 6), of these 26 were 28 weeks gestation or older and thus still meet the stillbirth definition. Six percent (*n* = 33) of observer-assessed stillbirths were under 28 weeks gestation at admission, most in Pokhara, NP (17.5%), 11 of these were 1000 g or more.

### Objective 3: Stillbirth classification

**Fig. 7:**
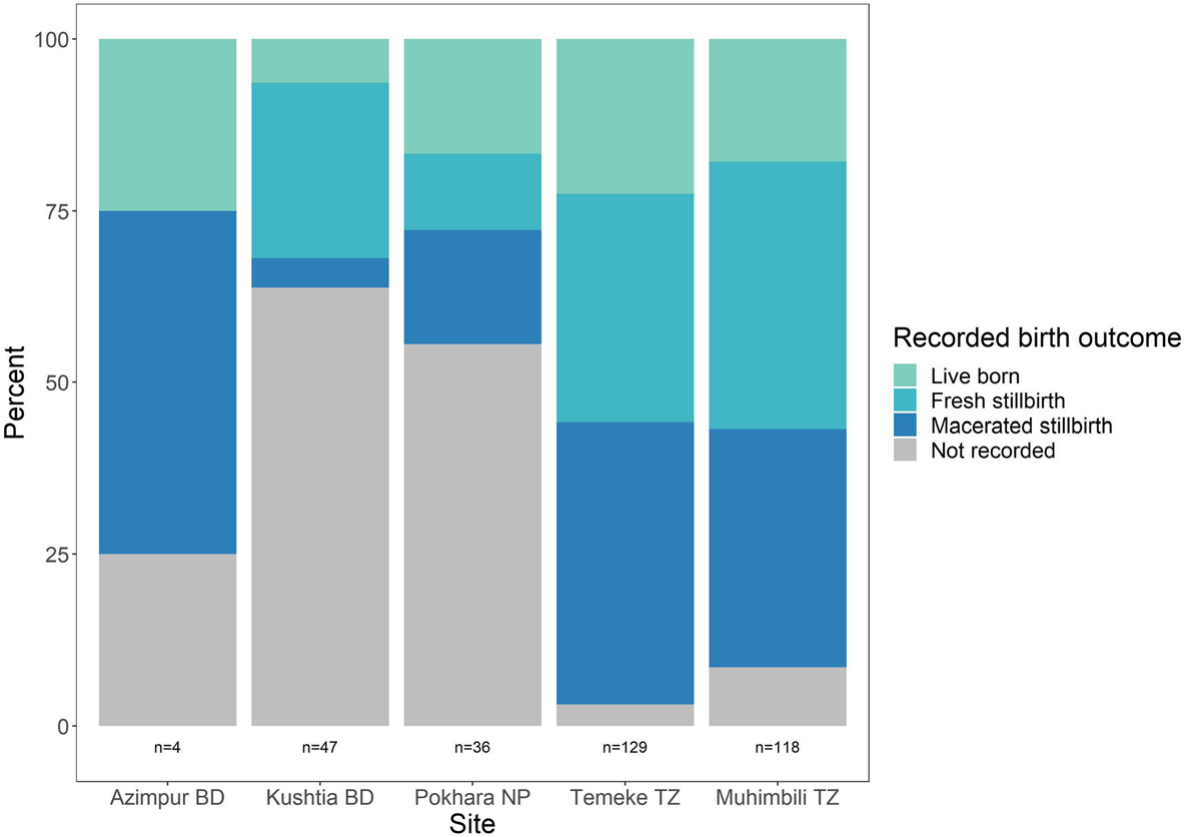
Register-recorded birth outcome for confirmed intrapartum stillbirths (*n* = 334), EN-BIRTH study. BD = Bangladesh, NP = Nepal, TZ = Tanzania

Across sites, 97.6% of women had their fetal heart rate checked at admission, however, 1.1% (*n* = 249) of women in the sample had no admission fetal heart rate recorded despite having the heart rate checked or the rate was recorded as absent. Only those with a recorded rate on admission who were later observed to be stillbirths were considered intrapartum stillbirths. While in Temeke, TZ, four in ten (41.0%) intrapartum stillbirths were recorded as macerated stillbirths, in Kushtia, BD fewer than 5% of intrapartum stillbirths were recorded as macerated stillbirths (Fig. 7).

### Objective 4: Gap analysis for coverage and measurement

**Fig. 8:**
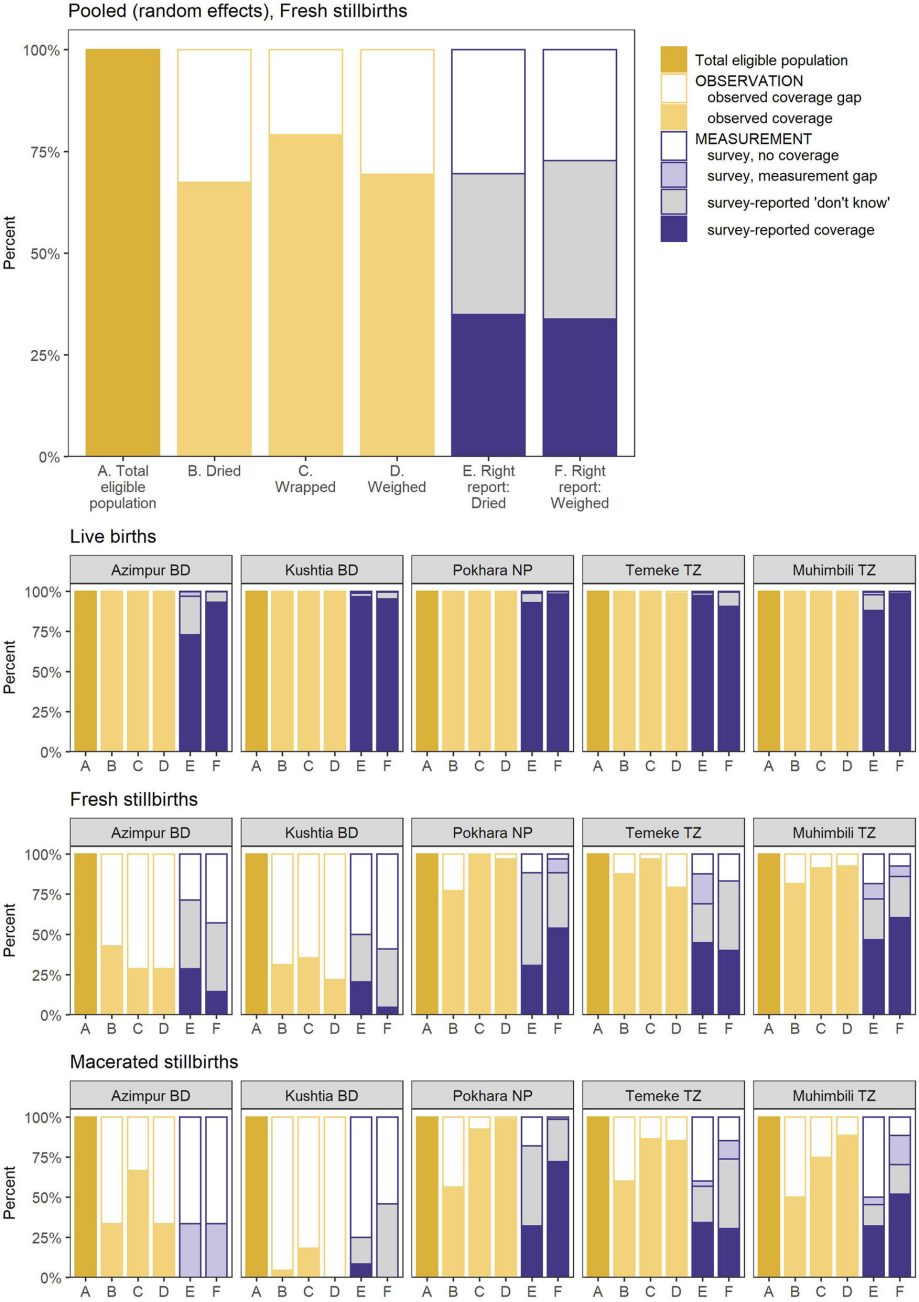
Gap analysis for coverage and measurement by birth outcome, EN-BIRTH study. Observed: live births (*n* = 22,464), fresh stillbirths (*n* = 230), macerated stillbirths (*n* = 277); Survey: live births (*n* = 20,050), fresh stillbirths (*n* = 157), macerated stillbirths (*n* = 200); BD = Bangladesh, NP = Nepal, TZ = Tanzania

Coverage of drying, wrapping, and weighing was very high (> 98%) for live births in all hospitals (Fig. 8). Coverage of these interventions was lower among stillbirths. Among fresh stillbirths in Nepal and Tanzania, over two-thirds were dried, wrapped, and weighed. In Bangladesh, however, less than half of fresh stillbirths were dried (31.3–42.9%), wrapped (28.6–35.5%), or weighed (21.9–28.6%). Among macerated stillbirths in Nepal and Tanzania, coverage of drying was lower (50.0–60.0%) while wrapping and weighing coverage was similar to fresh stillbirths (75% or more).

Survey-reported coverage of drying and weighing among women with live births was high and close to observed coverage. In Azimpur, BD, “don’t know” responses were high for survey-reported coverage of drying of live births but mostly among women giving birth by caesarean section. Among stillbirths, “don’t know” responses were higher, almost all above 25% and as high as 57.7% for survey-reported drying of fresh stillbirths in Pokhara, where observed coverage of drying was 77.1%.

### Objective 5: Barriers and enablers to routine documentation

#### Routine register design

Labour and delivery ward registers varied in design among the five hospitals; Nepal and Tanzania used the same registers during data collection, but Bangladesh registers were updated during the study as part of a national register standardisation programme unrelated to the EN-BIRTH study. We present results only from the revised national register. The updated Bangladesh registers had specific columns for newborn outcome: live birth or a column for stillbirth, subdivided into two columns for fresh and macerated stillbirths (Fig. 4). Instructions noted to tick for “yes” and leave blank for “no”. In Nepal, birth outcome was recorded in a blank box under a column titled “Outcome of baby/APGAR score”. No specific instructions were present to indicate type of stillbirth, but in practice the type of stillbirth was written instead of the APGAR score. In Tanzania, birth outcome was recorded in a blank box under a heading “Complications during pregnancy, labour and outcome of delivery and condition of mother and child”. A subheading for type of stillbirth was noted as “FSB/MSB” (fresh stillbirth/macerated stillbirth). In practice, a dash was noted in the box if the outcome was not a stillbirth and “FSB” or “MSB” was recorded to note the type of stillbirth.

#### Documentation practices in routine registers

The overall qualitative analysis for the EN-BIRTH study identified three categories surrounding barriers and enablers to routine recording of health information in hospital registers: 1) register filling 2) register use and 3) register design, where the data culture influences all of these aspects [27]. For register-recording of birth outcome, we found register use and register filling were enablers, whilst data culture acted as a barrier (Additional file 7). Respondents did not discuss register design in relation to recording birth outcomes.

##### Enabler - register filling

Supervision and support from senior nursing staff facilitated completeness and accuracy of birth outcome recording. This feedback reiterated to health workers that birth outcome recording is important and encouraged them to record it correctly.

“*If there is a neonatal death … that will be detailed … like all the staff sisters [senior nursing staff] will see whether it has been recorded or not … If one is writing and she finished properly but also other staff will come and check is it done […] but mostly if there are stillbirths then they will exactly record it properly*”.

-Data collector, Pokhara NP

##### Enabler - register use

Data demand was an enabler for accurate recording of birth outcomes as health workers understood the importance of tracking this information.

“*It is valuable because of this, how many patients are admitted in hospital, and how well those patients get service, and how many people are healthy, how many leaving alive, how many leaving dead. This document is very important in determining a rate. Again, the documentation is very important for those who come to the hospital whether they are getting proper service or not”.*

-Data collector, Kushtia BD

Participants associated register completion with broader aims of improving treatments and care for women and babies, which motivated their behaviours:

“*It must be done for the mother and child’s documentation because if anyone wants to know the cause of death information as well as their complications, how we managed these cases, delivery complications, referral information, outcome information (live/still birth) we can understand from here. […] if we do not keep documentation on outcomes how do we identify the success rate and treatment quality?*”

-Health worker, Kushtia BD

##### Barrier - data culture

The perceived need to document serious events such as stillbirths was recognised, but participants also expressed a hesitancy to record “more negative” outcomes such as neonatal deaths. This hesitancy could lead health workers to record neonatal deaths as stillbirths or abortions:

*“They always want us to record something good … avoid bad things […] Suppose, everything that I have is positive or good. That is the baby is well and also the mother, and everything is well. But, if there is anything bad within it, they do not want to record it. […] a child was born just with the arrival of the patient (mother), but the child was alive […] when the patient is taken to the ward, after some time it is reported that the baby has died. In that case, to do the report well, they take it as a problem. That’s why they have recorded it as 'abortion'.”*

-Data collector, Azimpur BD

## Discussion

Despite the magnitude of the issue, stillbirths lack visibility and accountability, often not counting in measurement systems. Valid data on stillbirths that is more widely and frequently available is essential to track the *Every Newborn* target of ending preventable stillbirths by 2030 [21] and improve care in facilities [29]. All three of these countries (Bangladesh, Nepal and Tanzania) have set a stillbirth reduction target [30]. EN-BIRTH is the largest study so far to test validity of the register-recorded stillbirth rate in hospitals in LMICs. We found hospital stillbirth rates accurately recorded in routine registers and included over 90% of the observed rate (2.9 per 1000 total births lower). We note that the overall study stillbirth rate of 23.8 per 1000 total births is lower than the true rate in these facilities, due to exclusion of women whose babies already had no heart rate on admission. Despite these selection criteria, we observed 550 stillbirths.

The register-recorded stillbirth rate in Pokhara, NP only under-estimated the observed rate by 1.7 per 1000 total births (ratio 0.9), despite being recorded in a non-specific column, being comparable with hospitals using registers with specific columns. The Pokhara hospital had a much smaller register, collecting 31 data points compared to 58 in Bangladesh and 45 in Tanzania [31]. Completeness of recording varied between the two sites in Bangladesh despite having identical register designs. While almost no birth outcomes were missing in the register in Azimpur (0.04% not recorded), 9.0% were not recorded in Kushtia. Data collectors rarely indicated data were not readable (0.03%), and percent agreement for double data extraction was high, however, there were lower inter-rater kappa results for register-recorded birth outcomes. More research is needed to improve data extraction as this is the first step for data flowing to higher levels in the health system. Registers and health information systems requiring only the critical data points decrease the burden on frontline health workers and improve reporting [32]. Additionally, supportive supervision and an enabling organisational context in Nepal may have contributed to improved accuracy.

For international comparison, stillbirth rates are measured using specific cut-offs of gestational age or birthweight [33]. Most stillbirths in this study (91%) were classified with correct cut-offs. Importantly, the majority of stillborn babies were weighed in most sites, however, birthweight was missing for almost one-third of stillbirths, mostly in Bangladesh. Since routine early pregnancy dating ultrasound is not yet widely available in LMICs [34], identifying accurate gestational age at birth remains challenging. EN-BIRTH data collection focused on labour ward admissions, which may account for the low number of observed early fetal losses (22 to 28 weeks’ gestation) as these cases are likely to have been referred elsewhere for care in these study settings. Research in India showed most women experienced early fetal loss at home [35].

Although there were a small number of neonatal deaths on the labour ward, we found more than half were misrecorded as stillbirths (21/36). This is in contrast to a much smaller percentage of stillbirths being misclassified as neonatal deaths (17/430) (Fig. 5). Research in India has shown that neonatal deaths within the first minutes of life were often recorded as stillbirths [36]. Flaccid newborns are often misclassified as fresh or intrapartum stillbirths since the clinical distinction can be difficult [37, 38], and is dependent on assessing the newborn heart rate at birth. Resuscitation practices influence misclassification [39]. Stillbirths and immediate neonatal deaths have decreased in settings where emphasis on the golden minute after birth and immediate resuscitation became standard [40–42].

We found stillbirth appearance (fresh/macerated) was not a good proxy for observed timing of stillbirth, resulting in underestimation of intrapartum stillbirths, consistent with another study which found provider description of fresh/macerated to be inaccurate compared to actual time since fetal death [43]. This results in a missed opportunity for quality improvement of facility-based intrapartum care, given many of these deaths could be preventable with timely access to quality midwifery and obstetric interventions [44, 45]. Factors such as maternal hyperthermia or sepsis, prolonged rupture of membranes, or bacterial infection may accelerate the skin appearance of “maceration” [43]. Current recommendations for routine collection of stillbirth data include disaggregation by fresh/macerated [46] or reporting an intrapartum or fresh stillbirth rate [47].

To capture true intrapartum stillbirth rates, standard of care must be to measure, record, and use the presence of fetal heart sounds on admission. Accountability for deaths after admission to labour ward requires a culture shift across all health system building blocks. Frontline health workers need supportive supervision, protocols, training and equipment such as fetal dopplers. A study in Tanzania showed that programmatically relevant timing of stillbirth data can be collected in routine registers with widespread use of Doppler devices to assess fetal heart rate on admission to maternity services [48]. Importantly regular fetal heart rate monitoring throughout labour and rapid response to abnormal rate or decelerations could prevent most intrapartum stillbirths, and yet is even more poorly done [49].

Drying, wrapping, and weighing of stillbirths may be a marker of respectful care for families with stillbirths where stillborns are handled gently and treated like babies. Whether the family were given the option to hold their stillborn baby and how the child was wrapped was not captured in EN-BIRTH. In high-income settings, families are usually given opportunity to spend time grieving with their stillborn child [50]. However a survey of health professionals around the world estimated that nearly three-quarters of women are not given the opportunity to hold their stillborn babies [9]. A systematic review of experiences of care after stillbirth in LMICs showed the importance of addressing barriers in the health system to improve provision of respectful care as well as women and staff’s desire for bereavement care [51]. Additionally, we examined women’s survey report if their stillborn babies were dried or wrapped to understand if the woman observed these events and could report on them; we found high levels of “don’t know” responses suggesting these babies may have been kept away from their mothers. More research is needed on such experiences and to inform locally appropriate bereavement packages for families, and support for health workers after stillbirths and neonatal deaths [52].

While the EN-BIRTH study included surveys of women’s report on exit from facility to explore survey-reported validity of measurement for maternal and newborn indicators, women were not asked about birth outcome. The EN-BIRTH tablet custom-built software was programmed to skip sensitive questions to minimise risk of further emotional trauma during interviews with women who experienced stillbirth [22]. In the development of the EN-BIRTH study, interviewing mothers of stillborn babies was raised as an ethical challenge. Parents of stillborn babies who formed part of the EN-BIRTH public involvement groups expressed that each mother should be able to choose or decline to be interviewed. In our sample, 385 mothers of stillborn babies out of 412 approached for interview consented to participate. The *Every Newborn-*INDEPTH multi-country study similarly investigated survey-reported data on stillbirth, including the feasibility of capturing information on timing of stillbirth [13, 53].

### Strengths and limitations

A strength of this study is the size, including over 23,000 births across five hospitals in three countries, and using observation as a gold standard to compare to register-recorded indicators. A user-friendly tablet with a custom-built data collection application was used to reduce delays or omissions in recording events. We looked at overall stillbirth recording, details for intrapartum stillbirths, and coverage of care for stillborn babies. Limitations of the present study should be noted. The stillbirths in this study are not representative of all stillbirths in these hospitals, selectively excluding sicker women unable to consent and specifically excluding antepartum stillbirths due to the study’s inclusion criteria of presence of a presumed positive fetal heart rate on admission. Our assessment of stillbirth classification (fresh/macerated) was only of observed intrapartum stillbirths and we were not able to assess classification in antepartum stillbirths. The gold standard used to identify stillbirths was observation of signs of life by trained clinical observers, but a true gold standard requires heart rate assessment immediately after birth [37]. The estimated gestational age used in this study was collected from patient records or the woman’s report at recruitment, which has variable accuracy. To detect effects from the presence of EN-BIRTH data collectors, we compared during-study register data to register data from the year prior to the study. Overall there were very few changes, apart from in Bangladesh where the register had changed during the study to a national standardised register [23].

## Conclusions

Our results show the validity and utility of facility register-recorded data to capture birth outcomes, however, we highlight challenges with identifying timing of stillbirth from appearance. Capturing stillbirths happening after facility admission using presence of fetal heart rate needs to be the standard of care. On admission to hospital in labour, every woman and her baby have the right to be assessed, including knowing if the baby is alive. Health workers require this information in order to provide appropriate care during labour and birth, especially given the unique medical, obstetric and psycho-social needs of women and families experiencing stillbirth. Every woman who experiences stillbirth has the right to know her stillborn baby’s weight and information about what happened, as well as the opportunity to see her baby. Leadership is needed to use data at all levels of the health system, including locally, to drive change in improving care to prevent stillbirths as well as count them.

## Supplementary information

**Additional file 1.** EN-BIRTH study sites—National mortality rates and hospital context.

**Additional file 2.** STROBE checklist of items that should be included in reports of observational studies.

**Additional file 3.** Respondents for focus group discussion and in-depth interviews for EN-BIRTH Study.

**Additional file 4.** EN-BIRTH data quality assurance for gold standard – double observation and data entry.

**Additional file 5.** Validity measures (AUC, IF, PPV, NPV) by hospital and mode of birth for EN-BIRTH Study.

**Additional file 6.** Two-way tables for observer-assessed and register-recorded birth outcome by hospital/mode of birth, EN-BIRTH study.

**Additional file 7.** Barriers and enablers to routine recording of birth outcomes in the EN-BIRTH study.

**Additional file 8.** Ethical approval of local institutional review boards, EN-BIRTH study.

## Data Availability

The datasets generated during and/or analysed during the current study are available on LSHTM Data Compass repository, https://datacompass.lshtm.ac.uk/955/.

## Abbreviations

AUC: Area under receiver operator curve
BD: Bangladesh
CIFF: Children’s Investment Fund Foundation
DHS: Demographic and Health Survey Program
ENAP: *Every Newborn* Action Plan now branded as *Every Newborn*
EN-BIRTH: *Every Newborn*-Birth Indicators Research Tracking in Hospitals study
FSB: Fresh stillbirth
HMIS: Health Management Information Systems
ICD-10: International Statistical Classification of Diseases and Related Health Problems 10th Revision
icddr,b: International Centre for Diarrheal Disease Research, Bangladesh
IF: Inflation factor
IHI: Ifakara Health Institute
INDEPTH: Network
LMIC: Low-Middle Income Country
LSHTM: London School of Hygiene & Tropical Medicine
MSB: Macerated stillbirth
MUHAS: Muhimbili University of Health and Allied Sciences
NP: Nepal
NPV: Negative predictive value
PPV: Positive predictive value
PRISM: Performance of Routine Information System Management
SDG: Sustainable Development Goal
TZ: Tanzania
UNICEF: United Nations International Children’s Emergency Fund
WHO: World Health Organization
