# Supplementary material for: Stillbirths including intrapartum timing: EN-BIRTH multi-country validation study
Source: BMC Pregnancy Childbirth. 2021 Mar 26;21(Suppl 1):226. doi: 10.1186/s12884-020-03238-7 (PMC7995570; doi:10.1186/s12884-020-03238-7)
Supplement: Supplementary file 7 — Additional file 7. Barriers and enablers to routine recording of birth outcomes in the EN-BIRTH study. [file 12884_2020_3238_MOESM7_ESM.pdf]

Every Newborn BIRTH multi-country validation study: informing measurement of coverage and quality of maternal and newborn care

Stillbirths including intrapartum timing: EN-BIRTH multi-country validation study

Additional File 7: Barriers and enablers to routine recording of birth outcomes, EN-BIRTH study

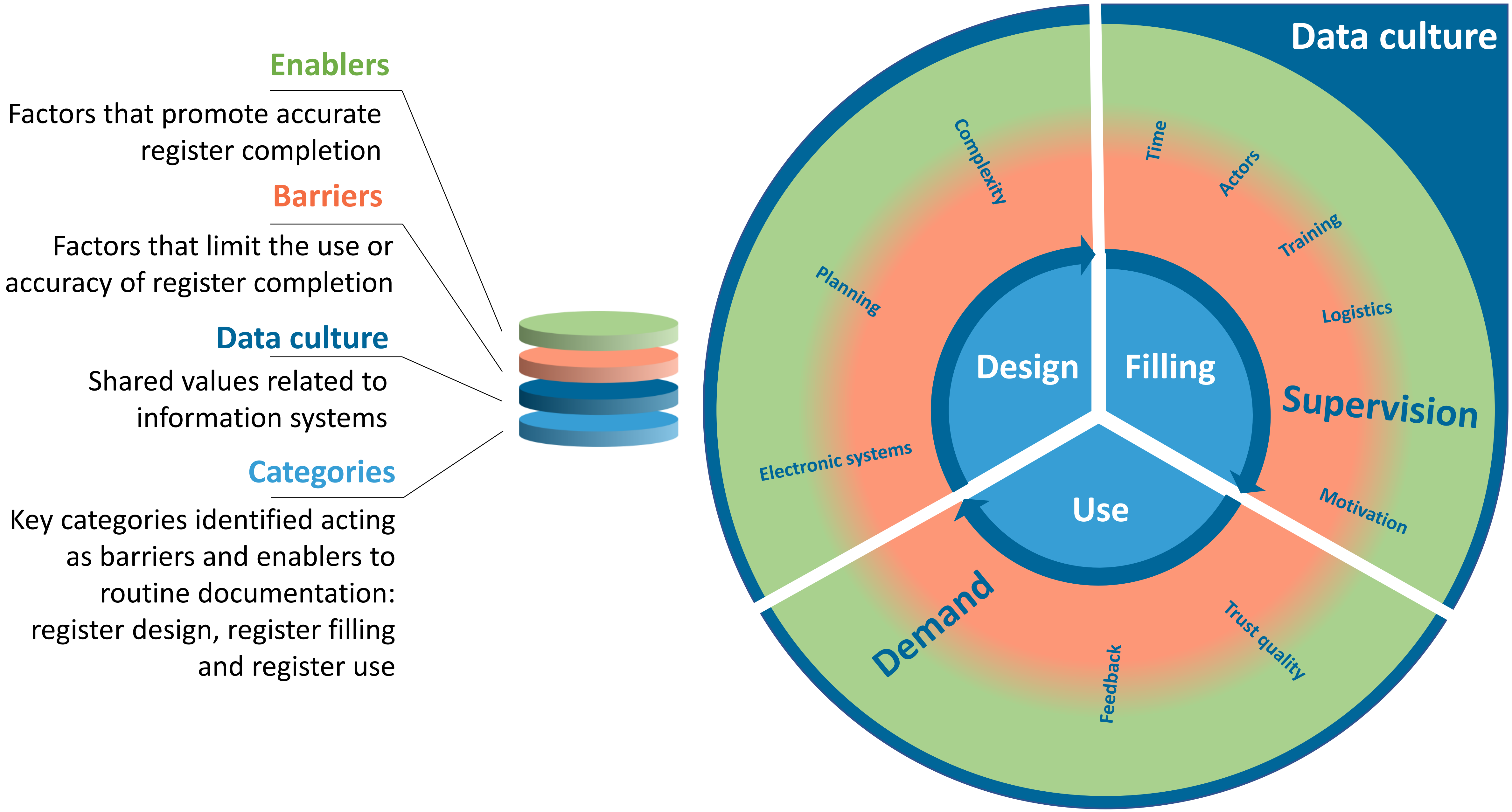

This figure illustrates the overall barriers and enablers to facility-based data collection identified by EN-BIRTH participants. The bold text are the issues specific to birth outcome. The transition from red to green is a reminder that most factors identified by participants could serve as either a barrier or enabling factor depending on the facility-level resources and management
